# Evaluation and Correlation Analysis of the Rheological Properties of Ground Tire Rubber and Styrene Butadiene Styrene Compound-Modified Asphalt

**DOI:** 10.3390/polym15153289

**Published:** 2023-08-03

**Authors:** Chunli Wu, Xiaoshu Tan, Liding Li, Chunyu Liang, Yongchao Zhao, Hanjun Li, Fuen Wang, Long Zhang

**Affiliations:** 1 College of Transportation, Jilin University, Changchun 130025, China; tanxs20@mails.jlu.edu.cn (X.T.); lild17@mails.jlu.edu.cn (L.L.); 2 Jilin China Railway Expressway Co., Ltd., Changchun 130052, China; 1593266955m@sina.cn (Y.Z.); lihanjun@crecg.com (H.L.); wangfuen@crecg.com (F.W.); 15590053555@163.com (L.Z.)

**Keywords:** compound-modified asphalt, ground tire rubber, rheological property, creep recovery property, 2S2P1D model, correlation analysis

## Abstract

With the increase in highway traffic volume, many waste tires are being produced, which puts serious pressure on the global ecological environment. Processing waste tires into powder and adding them to asphalt is an important and effective way to solve this noticeable environmental challenge. In this paper, to produce ground tire rubber (GTR) and styrene-butadiene-styrene (SBS) compound-modified asphalt, GTR was put into SBS-modified asphalt (GTRSA). Subsequently, some ordinary property tests, frequency sweep tests, and multiple stress creep recovery tests were conducted to investigate the conventional properties and rheological properties of GTRSA. Moreover, the 2S2P1D (two springs, two parabolic elements, and one dashpot) model was adopted to analyze the consequences of adding GTR content on the rheological properties of GTRSA. Finally, the Pearson correlation coefficient was employed to reveal the connection between the conventional properties and the rheological properties. The results show that GTR has a great impact on improving the rutting resistance, thermo-sensitive performance, shear resistance capability, stress sensitivity, and creep recovery performance of GTRSA. Adding 20% GTR can improve the creep recovery rate to 80.8%. The 5 °C ductility index suggests that GTR makes a difference to the low-temperature properties. The rheological properties and conventional properties had a strong linear link.

## 1. Introduction

**Figure 1:**
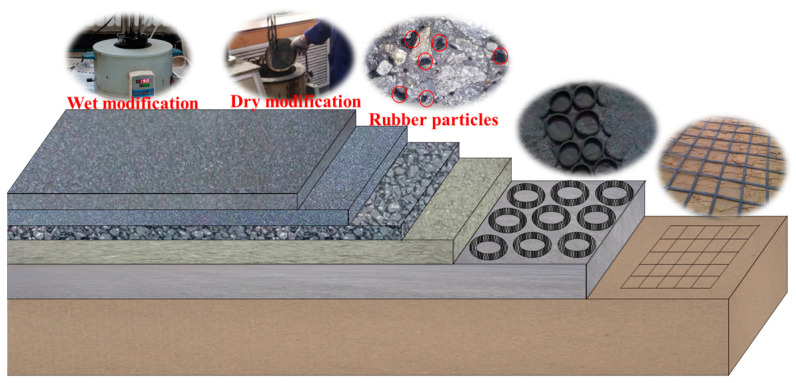
Application scheme of waste tires in road engineering.

In recent years, with the increase in vehicle ownership, a large number of waste tires have been produced [1]. These waste scrap tires occupy a large amount of arable land, and some incineration treatments also produce large amounts of harmful gases and CO_2_, which seriously endanger the natural environment [2]. In order to improve this situation, road engineering researchers have been utilizing waste tires in road construction [3]. At present, the application of waste tires in pavement construction takes on many forms: roadbed restraint with intact tires, reinforcement with tire strips and nets, toughening, and vibration damping with waste tire particles, as well as modification and enhancement with waste tire powder [4], as shown in Figure 1. Adding ground tire rubber (GTR) can significantly improve the high-temperature and low-temperature performance and the fatigue performance of asphalt binders [5]. However, with the deterioration of the natural environment and the increase in heavy traffic loads, the demand for asphalt binders with high viscosity is growing with each passing day. Therefore, compounding GTR and other materials to modify asphalt is also gradually being emphasized.

Researchers have proposed the modification of asphalt by combining inorganic materials such as carbon nanotubes [6], nano-SiO_2_ [7], graphene nanoplatelets [8], rice-husk ash [9], diatomite [10], and recycled glass powder [11] with GTR. Adding carbon nanotubes and nano-SiO_2_ into GTR-modified asphalt can enhance the elasticity and improve the resistance of the asphalt to creep [6,7]. In addition, nano-SiO_2_ can hinder UV radiation and enhance the resistance of the asphalt to UV aging [7]. The high- and low-temperature properties as well as the viscoelasticity of rubber-modified asphalt have successfully been enhanced by graphene nanoplatelets. Aside from that, the segregation of rubber-modified asphalt and the adhesive bonding between rubber-modified asphalt and aggregate have been optimized by adding graphene nanoplatelets [8]. Diatomite, recycled glass powder, and rice-husk ash can improve the high-temperature properties of matrix asphalt, but diatomite has a negative effect on the low-temperature properties of rubber-modified asphalt [9,10,11]. The road performance of asphalt can be improved and the asphalt material can gain some functionality by mixing inorganic components and GTR. However, these nano-inorganic materials are expensive to prepare and can significantly increase the asphalt content of corresponding mixtures.

In addition, there are also many studies compounding organic materials (such as low-density polyethylene (PE) [12], high-density PE [13], waste plastics [14], bio-oil [15,16], and SBS [17]) with GTR to modify and enhance asphalt properties. Ouyang et al. [12] showed that adding GTR can reduce the density difference between low-density PE and asphalt and improve the storage stability of the compound-modified asphalt. Gibreil et al. [13] reported that compound high-density PE and GTR can significantly strengthen the resistance to water damage and permanent deformation of asphalt mixtures. Zhang et al. [14] confirmed that asphalt modified by combining waste plastic with GTR exhibited excellent elastic recovery and presented optimal properties at 5% waste plastic and 15% rubber powder. Wang et al. [16] described that the addition of bio-oil to GTR-modified asphalt can enhance the workability of GTR-modified asphalt, but it has adverse effects on the high- and low-temperature properties as well as the fatigue properties. Gong et al. [17] reported that GTRSA has better resistance to aging and plastic deformation. Zhu et al. [18] emphasized that GTR and SBS-modified asphalt not only have excellent conventional properties, but also the interface bonding between the compound-modified asphalt and the aggregate is superior to that of SBS-modified asphalt. Liu et al. [19] analyzed the effect of low-content GTR on the properties of SBS-modified asphalt, and the study results pointed out that the plasticity of GTR and SBS-modified asphalt increased with a reduction in GTR content (from 2% to 6%), and the softening point and elastic recovery rate increased. Wang et al. [20] demonstrated that GTR and SBS-modified asphalt outperforms matrix asphalt in terms of high- and low-temperature characteristics and its viscosity increases significantly when the GTR content exceeds 15%. Zhou et al. [21] and Wu et al. [22] elaborated that the low-temperature properties of GTRSA are superior to those of SBS/TPS compound-modified asphalt and SBS-modified asphalt, adjusting to the needs of cold-climate paving materials. Yang et al. [23] introduced the concept that SBS/GTR composite-modified asphalt can develop a stable cross-linked structure, which significantly improves the storage stability of asphalt.

From the above studies, asphalt modified by compounding GTR and SBS will have better application prospects [24]. GTRSA possesses greater road performance than those asphalts combining GTR with inorganic or other organic materials. However, the most recent studies on the performance of GTRSA are mostly focused on its conventional pavement performance, and research on its rheological properties is still lacking. Furthermore, research on the relationship between conventional pavement performance and rheological performance is currently missing. Accordingly, a series of conventional tests, frequency sweep tests, and multiple stress creep recovery (MSCR) tests were conducted to evaluate the conventional properties and the rheological properties dependent on the loading frequency, test temperature, loading time, and loading stress in this study. The correlation between the conventional performance and the rheological performance was analyzed using the experimental results and the Pearson correlation coefficient. This study can give a theoretical foundation for the use of GTRSA in pavement engineering.

## 2. Materials and Methods

### 2.1. Materials

In this paper, GTRSA was produced by adding GTR into SBS-modified asphalt. The adding proportions of GTR were set as 5%, 10%, 15%, and 20% of the SBS-modified-asphalt weight, which was a compound-modified asphalt with high viscosity. According to the JTG E20-2011 specification, the basic performance indexes of the SBS-modified asphalt (4% SBS content, linear shape, S/B ratio of 4/6) are displayed in Table 1. The SBS-modified asphalt was a commercial modified asphalt from Panjin, Liaoning Province, China.

**Figure 2:**
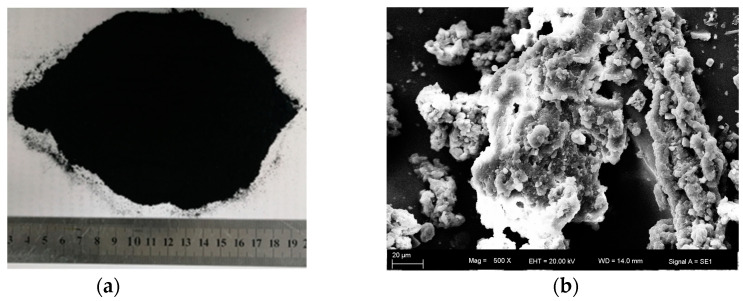
The macro- and micromorphologies (SEM) of the GTR: (**a**) macromorphology; (**b**) micromorphology (SEM).

The GTR used in this study was from Changchun, Jilin Province, as shown in Figure 2. Table 2 displays its basic performance indexes. The particle size of the GTR was 40 mesh. Following is how the GTRSA was produced [25,26]: first of all, the SBS-modified asphalt was melted to a flowing state at 170 ± 5 °C; secondly, the GTR was blended into the melted SBS-modified asphalt [20], and the modified temperature was then raised to 190 ± 5 °C and the sheared velocity was set at 3000 rpm for 40 min; subsequently, the compound-modified asphalt was then stirred at 1000 rpm for 20 min; finally, the stirred GTRSA was swollen at 175 ± 5 °C for 60 min.

### 2.2. Research Methods, Experimental Methods and Analytical Theories

#### 2.2.1. Research Methods

The main methods of this research are as follows:

1. Implement penetration tests, cone penetration tests, ductility tests, softening point tests, and viscosity tests to analyze the conventional properties of GTRSA;
2. Perform frequency sweep tests and MSCR tests to quantitatively analyze the viscoelastic characteristics of GTRSA according to rutting factor, fatigue factor, 2S2P1D model, elastic recovery rate, and unrecoverable creep compliance;
3. Apply the Pearson correlation coefficient to compute the correlation between the conventional performance and the rheological performance of GTRSA and analyze the modification mechanisms of GTRSA based on the relevant results.

**Figure 3:**
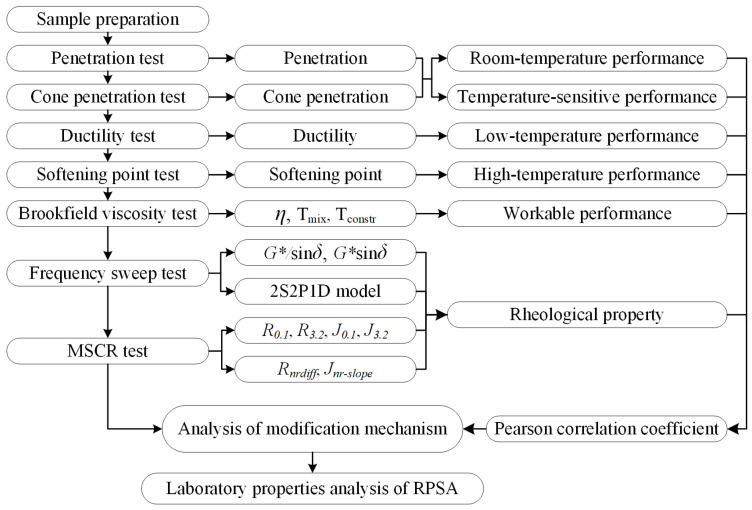
The research protocol of rheological properties and correlation analysis for GTRSA.

Based on these research methods, the research protocol of this paper was designed as shown in Figure 3.

#### 2.2.2. Experimental Methods and Analytical Theories

- Conventional performance tests

**Figure 4:**
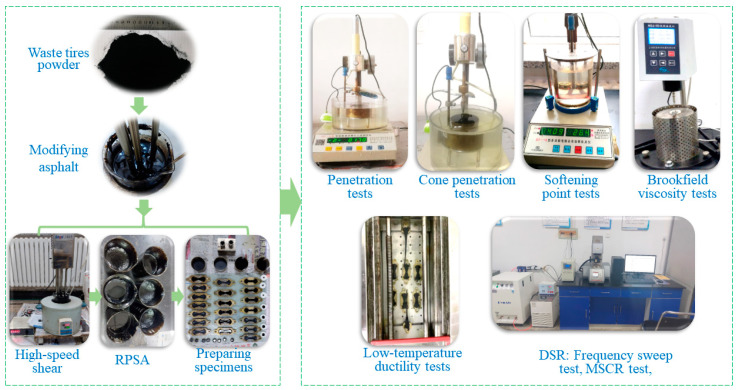
Preparation and mechanical performance testing of GTRSA.

According to the JTG E20-2011 specification [27], penetration tests, softening point tests, ductility tests at 5 °C, and Brookfield viscosity tests were carried out to evaluate the conventional pavement performance of GTRSA. Moreover, in order to make up for the deficiency of the penetration test in assessing the modified asphalt performance, Cone penetration tests were also used to assess the intermediate temperature performance of GTRSA. The experimental procedure and laboratory sample are shown in Figure 4.

- Frequency sweep test

On the basis of ASTM D7175 [28], the complex shear modulus of GTRSA was tested ranging from 58 °C to 88 °C at 6 °C increments. A 25 mm diameter parallel plate with 1 mm height was used in this study. The range of the sweep frequency was set from 0.1 Hz to 20 Hz. In addition, a 1% loading strain was adopted throughout the frequency sweep test, which ensured that the asphalt deformation kept in its linear viscoelastic state.

- MSCR test

On the basis of ASTM D7405 [29], MSCR tests of GTRSA were conducted at high temperatures (70 °C). A stress loading mode was applied, and the two stress loading levels were set as 0.1 kPa and 3.2 kPa, respectively. The average percent recovery (*R*_0.1_ and *R*_3.2_) and the average non-recoverable creep compliance (*J_nr_*_0.1_ and *J_nr_*_3.2_) were computed using the test results and Equations (1)–(6) [30]:

(1) $$R_{0.1}=\frac{1}{10}\sum_{i=11}^{20}\frac{100\times (\varepsilon_{c}^{i}-\varepsilon_{r}^{i})}{\left(\varepsilon_{c}^{i}-\varepsilon_{0}^{i}\right)}$$

(2) $$R_{3.2}=\frac{1}{10}\sum_{i=21}^{30}\frac{100\times (\varepsilon_{c}^{i}-\varepsilon_{r}^{i})}{\left(\varepsilon_{c}^{i}-\varepsilon_{0}^{i}\right)}$$

(3) $$J_{nr0.1}=\frac{1}{10}\sum_{i=11}^{20}\frac{\left(\varepsilon_{r}^{i}-\varepsilon_{0}^{i}\right)}{0.1}$$

(4) $$J_{nr3.2}=\frac{1}{10}\sum_{i=21}^{30}\frac{\left(\varepsilon_{r}^{i}-\varepsilon_{0}^{i}\right)}{3.2}$$

(5) $$R_{diff}=(R_{0.1}-R_{3.2})\times 100/R_{0.1}$$

(6) $$J_{\mathrm{nrslope}}=(J_{nr0.1}-J_{nr3.2})\times 100/3.1$$

where $\varepsilon_{0}^{i}$ and $\varepsilon_{c}^{i}$ are the initial creep strain and final creep strain, respectively, at the *i*-th loading cycle; $\varepsilon_{r}^{i}$ is the final strain after creep recovery at the *i*-th loading cycle; and *R_diff_* and *J_nrslope_* are the percent difference in recovery and in non-recoverable creep compliance between 0.1 kPa and 3.2 kPa, respectively.

## 3. Results and Discussion

### 3.1. Conventional Pavement Properties

#### 3.1.1. Intermediate Temperature Performance

**Figure 5:**
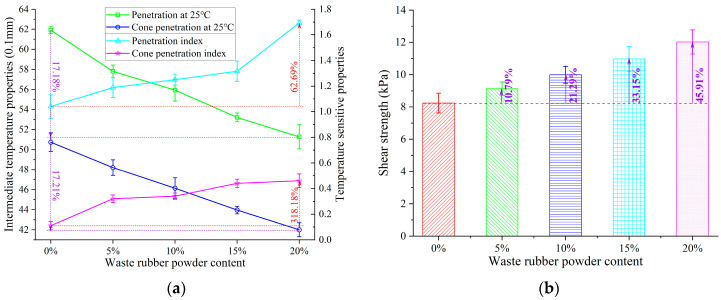
Performance indexes of GTRSA at intermediate temperature: (**a**) intermediate-temperature performance; (**b**) shear strength based on cone penetration.

Penetration and cone penetration at 25 °C were used to assess the impact of GTR content on the intermediate-temperature properties of SBS-modified asphalt, as shown in Figure 5. The penetration and cone penetration of GTRSA showed a decreasing trend with the increase in GTR content. After adding 20% GTR, the penetration and cone penetration decreased by 17.18% and 17.21%, respectively. Further, the shear strength *τ_c_* of GTRSA was calculated according to the cone penetration and Equation (7) [31], as shown in Figure 5b. The shear strength of GTRSA increased continuously with the addition of GTR, and the shear strength was increased by 45.91% after the addition of 20% GTR. This indicates that adding GTR can enhance SBS-modified asphalt’s cohesiveness at intermediate temperatures, which in turn enhances SBS-modified asphalt’s resistance to rutting at intermediate temperatures.

Temperature sensitivity is an important property that can be employed to assess how temperature affects GTRSA properties. In this paper, the penetration index (PI) and cone penetration index (CPI) were used to assess the temperature sensitivity of GTRSA at intermediate temperatures, and the CPI was calculated based on the calculating method of the PI (as shown in Equation (8)). The calculation results of the PI and CPI are shown in Figure 5a. The PI and CPI of GTRSA increased significantly with the increase in GTR, which indicates that the temperature sensitivity of SBS-modified asphalt can be further improved by adding GTR. The calculation results in Figure 5a show that the quantitative improvement of the PI and CPI was highly different, mainly due to the fact that the temperature sensitivity index was defined as a value of around zero, which is not suitable for quantitative assessment of the change in asphalt temperature sensitivity.

After adding rubber powder to asphalt, it will undergo swelling and partial dissolution under high-speed shear loads [3,32]. The swelled rubber powder can adsorb lightweight components (aromatics and saturates) in the asphalt [23,33], resulting in an increase in the relative content of resin and asphaltene in the compound-modified asphalt, and improving the asphalt’s shear deformation resistance. In addition, the partially dissolved crumb rubber can wrap around the crumb rubber microparticles, forming a layer of gel film on the surface of the crumb rubber particles. The rubber powder particles can be bonded through the gel film, forming a viscous semisolid continuous phase system, thereby increasing the viscosity resistance of the composite-modified asphalt to shear deformation and improving the shear deformation resistance of the composite-modified asphalt. Equations (7) and (8) are as follows:

(7) $$\tau_{c}=\frac{981Q\cos^{2}(\alpha /2)}{\pi h^{2}\tan(\alpha /2)}$$

(8) $$PI=\frac{30}{1+50A}-10$$

where $\tau_{c}$ is the shear strength, Pa; Q is the total mass of the cone needle, connecting rod and weight, 195g; h is the cone penetration, mm; α is the angle of the cone needle tip, 30°; A is the slope of the penetration or cone penetration with the test temperature.

#### 3.1.2. Performance at Low Temperatures

**Figure 6:**
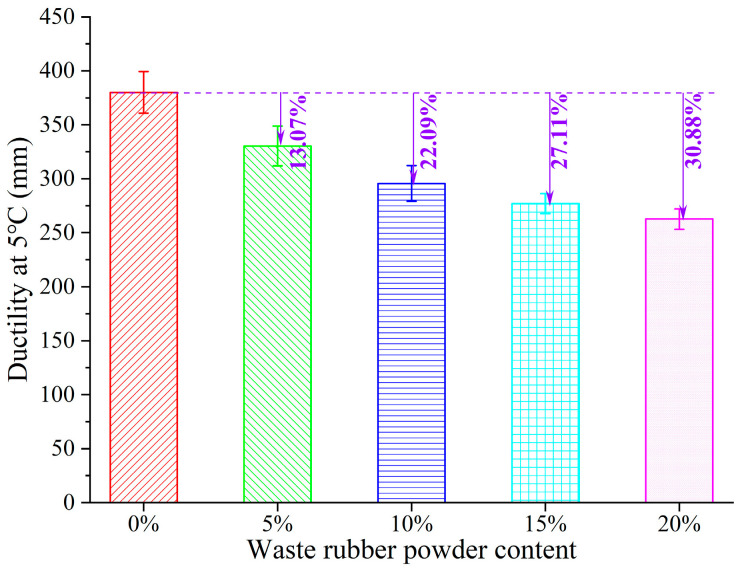
Low-temperature ductility of GTRSA.

In order to assess GTRSA’s low-temperature performance, its ductility at 5 °C was used. Figure 6 depicts the variations in low-temperature ductility with the GTR content. Adding GTR decreased the ductility of SBS-modified asphalt, and a 20% GTR addition reduced the ductility of SBS-modified asphalt by 30.88%, which implies that the addition of GTR can weaken the tensile ductility of GTRSA at low temperatures. This may be attributed to the addition of GTR microparticles to form a weak area in the tensile process and weaken the deformable capacity of GTRSA at low temperatures.

#### 3.1.3. High-Temperature Performance

**Figure 7:**
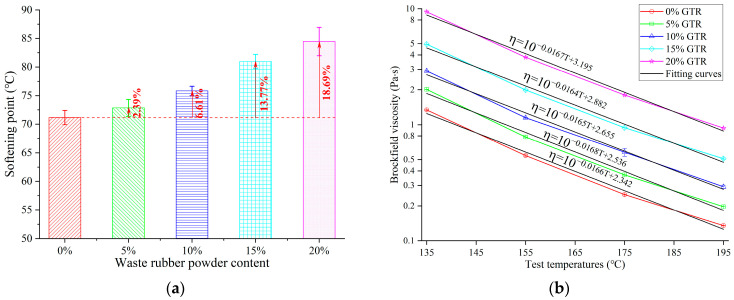
High-temperature performance index of GTRSA: (**a**) softening point; (**b**) Brookfield viscosity.

Tests of viscosity and softening point were used to examine GTRSA’s performance at high temperatures. The variation in the softening point and different temperatures of Brookfield viscosity with the content of GTR are depicted in Figure 7. The GTRSA’s softening point showed a rising trend with the increase in GTR. The softening point increased by 2.39%, 6.61%, 13.77%, and 18.69% after adding 5%, 10%, 15%, and 20% of GTR, respectively.

In addition, the Brookfield viscosity of GTRSA at different temperatures showed a negative exponential decrease with the increase in temperature. Increasing the GTR content obviously raised the Brookfield viscosity and softening point of GTRSA. From the softening point and Brookfield viscosity results, the high-temperature performance of GTRSA can be considerably improved by including GTR in SBS-modified asphalt, and the related asphalt mixture’s rutting resistance can be strengthened, which will be suitable for the construction of pavements in tropical areas.

#### 3.1.4. Workable Performance

**Figure 8:**
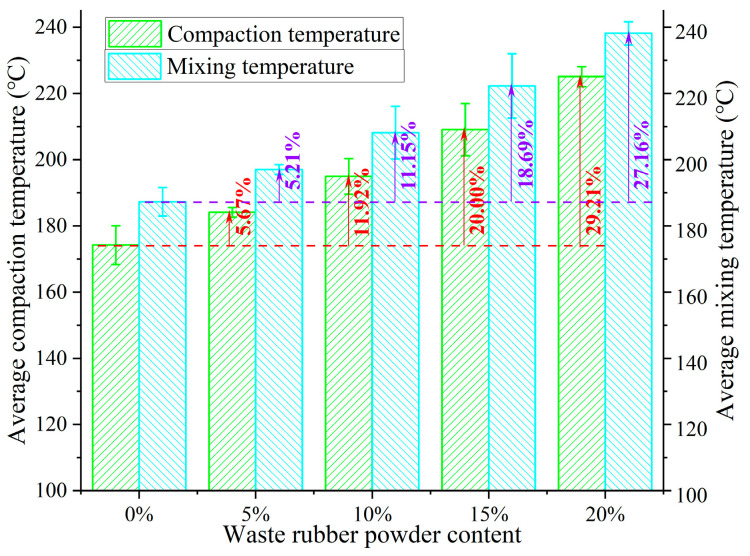
Compaction temperature and mixing temperature of GTRSA.

Workable performance is an important indicator of the practical applicability for asphalt, and excellent workability is beneficial to the mixing and compaction of asphalt pavement. In this research, the mixing and compaction temperatures were calculated based on the viscosity–temperature curve of Brookfield viscosity, as shown in Figure 7b. Although raising the Brookfield viscosity in the above study was able to characterize the improvement of the high-temperature performance of GTRSA, the corresponding mixing and compaction temperatures were also raised, as shown in Figure 8, which is not conducive to the construction of GTRSA. Therefore, adding GTR has a detrimental impact on the workable properties of SBS-modified asphalt. The mixing temperature and compaction temperature of the GTRSA increased by 29.21% and 27.16%, respectively, after the addition of 20% GTR.

According to the conventional performance of GTRSA, the additional content of GTR is recommended at 10~20%. In this range, it can be considered to appropriately reduce the content of GTR in cold areas and appropriately increase the content of GTR in hot areas.

### 3.2. Rheological Properties of GTRSA

#### 3.2.1. Impact of GTR Content on the Rheological Performance of Asphalt

**Figure 9:**
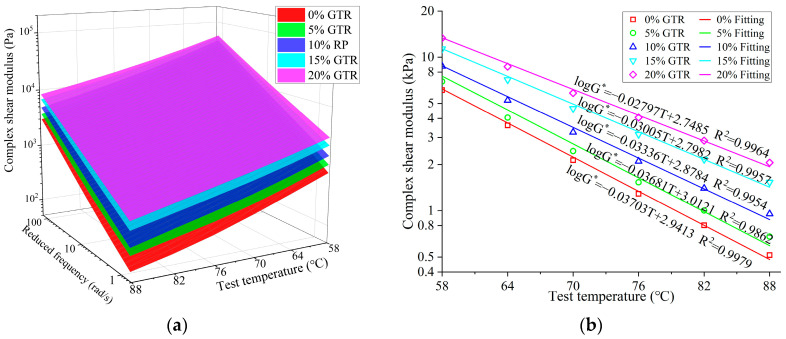
Complex shear modulus of GTRSA at different temperatures: (**a**) G* versus temperatures and frequencies; (**b**) G* versus temperatures.

The frequency sweep test was employed to obtain the variability of complex modulus (G*) for GTRSA with loading frequencies at different temperatures, as shown in Figure 9a. With the increasing of the temperature, the complex modulus of GTRSA showed a decreasing trend, because the shear resistance of asphalt reduces with increasing temperature. With an increase in frequency, the complex modulus of GTRSA showed a rising trend of GTRSA. This is primarily attributable to the increase in the viscous resistance of GTRSA following the increase in shear rate. In addition, the complex shear modulus of GTRSA at various frequencies and temperatures increased continuously with the increase in GTR content. Adding GTR can significantly enhance the GTRSA’s shear deformation resistance, which is beneficial to elevating the rheological properties of GTRSA. 

Figure 9b depicts the fluctuation of complex shear modulus at 10 rad/s with test temperatures, and the complex shear modulus of GTRSA shows a logarithmic decreasing trend with the increase in temperature. The fitting findings show that the slope of the fit decreased with increasing GTR content. Adding GTR can greatly weaken the SBS-modified asphalt’s temperature sensitivity at high temperatures; the higher the content, the more obvious the improvement.

**Figure 10:**
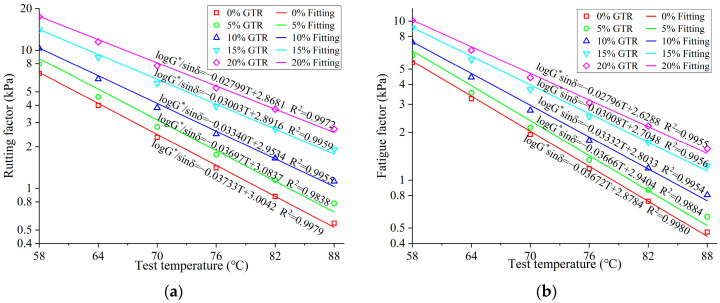
Rutting factor and fatigue factor versus test temperatures: (**a**) rutting factor; (**b**) fatigue factor.

The rutting factor (G*/sinδ) and fatigue factor (G*∙sinδ) of GTRSA were determined under various temperatures to further analyze the effect of GTR content on resistance to rutting and fatigue, as shown in Figure 10. The rise in the rutting factor and the decrease in the fatigue factor imply the improvement of the rutting and fatigue resistance of the asphalt binder, respectively [34]. From Figure 10, the G*/sinδ and G*∙sinδ of GTRSA decreased significantly with the increasing temperature. The exponential function was adopted to fit the curves. According to the fitting results, the attenuation of the rutting factor and fatigue factor with the temperature for GTRSA met the negative exponential trend well. The results demonstrate the deterioration of the asphalt’s rutting resistance with increasing temperature, but this does not mean there was improvement of the fatigue resistance. As the test temperature increased, the complex shear modulus decreased, and the rutting factor related to that decreased, which characterized the improvement of shear resistance and rutting resistance. However, the decrease in the fatigue factor caused by the reduction in the complex modulus did not reflect the increase in the elastic component determined by the phase angle. In reality, temperature increases resulted in a rise in the viscous component, a decrease in the elastic component, and a weakening of the fatigue resistance. Therefore, the fatigue factor is not suitable for evaluating the temperature’s impact on GTRSA’s fatigue performance.

Similarly, the additional GTR elevated the SBS-modified asphalt rutting factor; the bigger the improvement, the higher the extra content, which means that the additional GTR was able to considerably enhance the rutting resistance of GTRSA. From Figure 10b, adding GTR raised the fatigue factor of SBS-modified asphalt. The physical adsorption of GTR on light components of asphalt increased the complex shear modulus of SBS-modified asphalt, which caused the increase in the fatigue factor, but this does not represent the weakening of the fatigue properties of GTRSA. 

The variations in the rutting factor and fatigue factor with the temperature in semi-logarithmic coordinates were fitted, and the fitting results are shown in Figure 10. The fitting slope shows that the temperature sensitivity of the GTRSA decreased due to the rise in GTR content. The temperature sensitivity of the GTRSA was reduced by 23.86~25.02% after adding 20% GTR by using the fitted slope as an evaluation index.

#### 3.2.2. Rheological Property Analysis Based on the 2S2P1D Model

**Figure 11:**
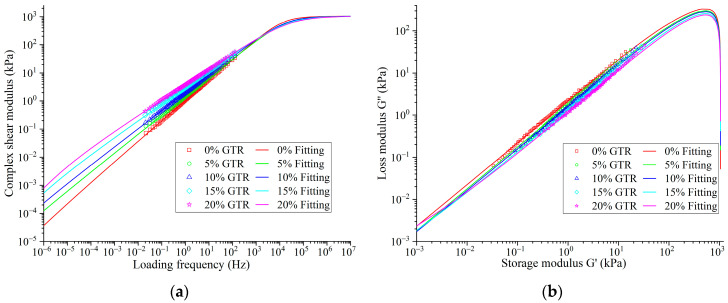
Master curves of construction and fitting analysis: (**a**) G* master curve fitting result; (**b**) Cole–Cole curves.

To assess how GTR content affects the rheological properties of SBS-modified asphalt, the time–temperature superposition principle and the Arrhenius equation (as shown in Equation (9)) were used to construct the complex modulus master curve for GTRSA at 58 °C. The outcomes are displayed in Figure 11a. The master curve was able to obtain the variant of the complex shear modulus of GTRSA over a wide frequency range, which was beneficial to obtain accurate fitting results for the viscoelastic model.

Subsequently, the 2S2P1D model (as shown in Equation (10)) was employed to fit these master curves. There were nine parameters to be fitted in the 2S2P1D model of Equation (10), and too many parameters seriously affected the reliability of the fitted results. According to the findings of the relevant literature [35,36], parameters that were not sensitive and do not affect the analysis were set as constants, and the setting results are shown as Gm, G0, and τ in Table 3. The parameters of the 2S2P1D model were calculated in Excel using the optimization approach, and their fitting results are displayed in Table 3. Figure 11’s master curves and the Cole–Cole curves show that the 2S2P1D model can reflect the rheological properties of GTRSA versus the loading frequency well.

The fitted parameters of fractional-order exponents k and h had an obvious decreasing trend with the increase in GTR content, which was clearly reflected in the slope of the Cole–Cole curves. And it indicates that with an increase in GTR concentration, GTRSA’s elasticity rises, which is advantageous for deformation recovery. From the fitting results of the Arrhenius equation, the activation energy Δ*E_a_* of GTRSA increased with the increase in GTR content, which means that the required deformation energy under the same shear strain increases continuously with the growth of GTR content. The shear deformation resistance of GTRSA is improved by the addition of more GTR. Equations (9) and (10) are as follows:

(9) $$\log(\alpha_{T})=\frac{\Delta E_{a}}{2.303R}\left(\frac{1}{T+273.15}-\frac{1}{T_{ref}+273.15}\right)$$

(10) $$G^{*}(j\omega )=G_{0}+\frac{G_{m}-G_{0}}{1+\mu {\left(j\omega \tau \right)}^{-k}+{\left(j\omega \tau \right)}^{-h}+{\left(j\omega \tau \beta \right)}^{-1}}$$

where *G_m_* and *G_0_* are the glassy shear modulus and static modulus of GTRSA; *G_0_* = 0. *λ*, *β*, and γ are the correlation coefficients of asphalt binder; and *ω* is the angular frequency, *ω* = 2π*f*.

### 3.3. GTRSA’s Creep Recovery Characteristics

#### 3.3.1. Results of MSCR Testing

**Figure 12:**
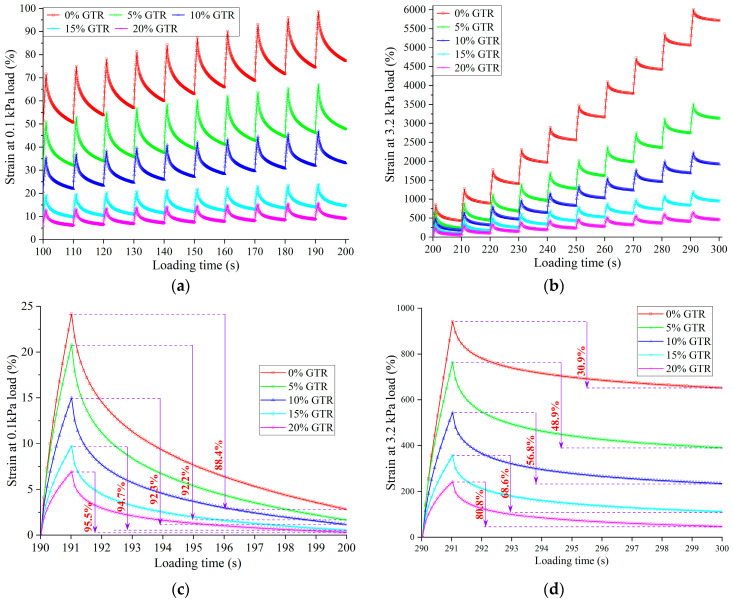
MSCR results of GTRSA at low and high stress: (**a**) creep and recovery at low stress; (**b**) creep and recovery at high stress; (**c**) creep recovery ratio at low stress; (**d**) creep recovery ratio at high stress.

MSCR experiments were performed to evaluate the GTRSA’s creep and recovery properties under different stresses in order to quantitatively study the improvement of GTR content on those qualities [37]. Figure 12 shows the creep and recovery of GTRSA in low- and high-stress situations. From Figure 12a,b, the inclusion of GTR was able to significantly decrease the shear creep deformation of GTRSA at high and low stress. The absorption and swelling of GTR in SBS-modified asphalt significantly raised the relative content of asphaltenes and resin in GTRSA and then reinforced the viscous resistance to shear deformation for GTRSA. Therefore, this implies that adding GTR can remarkably enhance the shear deformation resistance of GTRSA.

Figure 12c,d calculates the creep recovery rate of GTRSA for a single loading cycle at high and low stress. The calculation results indicate that SBS-modified asphalt itself had an excellent creep recovery rate at low stress, reaching 88.4%, while the creep recovery rate reduced dramatically at high stress, at only 30.9%. Large plastic damage deformation of SBS-modified asphalt developed at high stress, at which damage deformation is not recoverable, whereas adding GTR obviously promoted the SBS-modified asphalt’s creep recovery, particularly when stressed. The creep recovery rate was raised by 7.1 percentage points at low stress by the addition of 20% GTR and by 49.9 percentage points at high stress, reaching 80.8%. After adding rubber powder to SBS-modified asphalt, on the one hand, it was able to adsorb small molecular groups in the asphalt through shear swelling, increase the relative content of asphaltene and resin in the asphalt, and improve the viscosity resistance of the asphalt. On the other hand, rubber powder microparticles can only partially dissolve in asphalt, and the elastic cores of undissolved rubber powder microparticles were widely dispersed in the asphalt medium. During the loading process, the rubber powder micro-elastics caused a large number of shear bands and “silver lines” in the SBS-modified asphalt matrix, and the initiation and expansion of shear bands and “silver lines” consumed a lot of energy. Moreover, rubber powder microparticles with excellent elastic properties were also able to inhibit the growth and fracture of individual “silver lines”, delay the generation of destructive cracks in SBS-modified asphalt matrix, and improve the shear deformation resistance and creep recovery ability of composite-modified asphalt binder.

#### 3.3.2. Recovery from Creep and Resistance to Unrecoverable Creep Deformation

**Figure 13:**
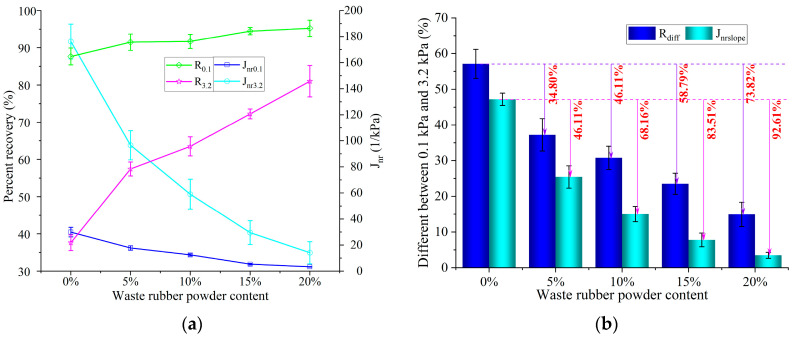
Creep deformation recovery of GTRSA at low and high stress: (**a**) percent recovery and non-recoverable creep compliance; (**b**) the stress sensitivity of GTRSA.

The percent recovery (*R*_0.1_ and *R*_3.2_) and non-recoverable creep compliance (*J_nr_*_0.1_ and *J_nr_*_3.2_) of GTRSA at low and high stress were obtained using Equations (1)–(6), as shown in Figure 13, to quantitatively investigate the creep recovery features of GTRSA. They showed an ongoing upward trend with the rise of GTR content, and *R*_0.1_ and *R*_3.2_ exhibited an ongoing rising trend. Under high stress, GTRSA’s creep recovery capacity improved more than it did under low stress. This is mostly due to the viscoplastic deformation produced in the SBS-modified asphalt, and the plastic damage led to a lower elastic recovery rate (of roughly 30%). And the shear deformation resistance of GTRSA was enhanced with the increase in GTR content, which means that under high stress, GTRSA with a high GTR content produces less creep deformation and viscoplastic damage deformation. Thus, GTRSA has better elastic recovery than SBS-modified asphalt. The non-recoverable creep compliance (*J_nr_*_0.1_ and *J_nr_*_3.2_) of GTRSA indicates that it has better creep recovery ability under low and high stress than that of SBS-modified asphalt. Then again, the large non-recoverable creep compliance of GTRSA under high stress represents a large non-recoverable damage deformation, indicating that it has less elastic recovery ability compared to low stress. High-stress-induced viscoplastic damage is mostly responsible for this.

Figure 13b shows the difference in the percentages of non-recoverable and recovery creep compliance between low and high levels of stress. The percentage recovery (*R_nrdiff_* and *J_nrslope_*) difference for SBS-modified asphalt is clear, indicating that the SBS-modified asphalt is sensitive to stress and prone to rutting deformation. By adding GTR, the stress sensitivity of SBS-modified asphalt can be greatly enhanced, and the enhancement becomes more noticeable as the GTR content increases. It can be seen from *R_nrdiff_* and *J_nrslope_* that the stress sensitivity of the asphalt binders can be increased by 73.82% and 93.89%, respectively, by adding 20% GTR.

### 3.4. Modified Mechanism and Correlation Analysis of GTRSA

**Figure 14:**
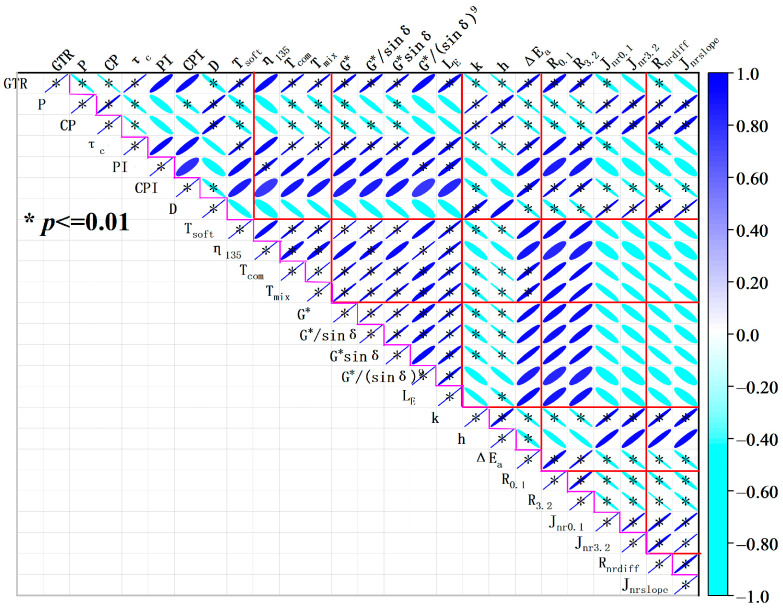
Correlation analysis results between the performance indexes.

In order to examine the relationship between the rheological and conventional properties of GTRSA, the Pearson correlation coefficient was used to determine the correlation between various indicators [38], as illustrated in Figure 14.

The results of the correlation coefficient showed that the frequency sweep test and MSCR test results of GTRSA were closely related to the GTR content. In addition, the frequency sweep test results were closely related to the softening point (*T_soft_*), viscosity (*η*_135_), mixing temperature (*T_mix_*), and compaction temperature (*T_com_*) of GTRSA. This is mainly attributed to the fact that the adsorption and inter-lap effect of GTR significantly improved the cohesion of GTRSA, which in turn improved the softening point, viscosity, and rutting resistance of the GTRSA. 

Moreover, the creep recovery and non-recoverable creep compliance of GTRSA were closely related to the indexes of the GTR content, penetration (P), cone penetration (CP), CPI, and ductility (D), as well as the 2S2P1D model parameters (k). The cohesion and elasticity of GTRSA were enhanced with the increase in GTR, resulting in the decrease in penetration and cone penetration of the GTRSA, and then the decrease in k, *J_nr_*_0.1_, as well as *J_nr_*_3.2_ and the increase in *R*_0.1_ and *R*_3.2_ were reflected in the rheological properties. In addition, the six types of rheological indexes of MSCR were highly correlated with each other, which also represents the close correlation between the rheological property indexes of GTRSA and reveals that they can be derived from each other by certain functions. In contrast, the relationship between the frequency sweep test outcomes and the MSCR test outcomes showed a weak linear correlation, which implies that a linear function may not be applicable in constructing the relationship between the frequency sweep test results and the MSCR test results.

## 4. Conclusions

- Adding GTR can significantly improve the anti-rutting deformation and temperature sensitivity at high and intermediate temperatures. This is mainly attributed to the shear swelling and partial dissolution of rubber powder in SBS-modified asphalt, which adsorbs the lightweight components (aromatics and saturates) in the asphalt, resulting in an increase in the relative content of resin and asphaltene in the GTRSA and improving the asphalt’s shear deformation resistance. However, GTR can have a negative effect on low-temperature performance and workable performance. Considering the conventional performance of GTRSA, the additional content of GTR is recommended at 10~20%;
- The elasticity of SBS-modified asphalt can be enhanced by adopting GTR as a compound modifier according to the fitted results of fractional-order exponents k and h. Due to GTRSA’s high activation energy Δ*E_a_*, the inclusion of GTR helps to increase the substance’s ability to withstand shear deformation;
- An increase of 20% GTR was able to increase the creep recovery rate by 7.1 percentage points at low stress and by 49.9 percentage points at high stress, reaching 80.8%. Moreover, by including GTR, SBS-modified asphalt’s stress sensitivity significantly increased. The addition of 20% GTR was able to increase the stress sensitivity of GTRSA by 73.82% and 93.89% according to the results of *R_nrdiff_* and *J_nrslope_*, respectively. The partially undissolved rubber powder microparticle elastic core was widely dispersed in the asphalt medium, which cannot be ignored in improving the creep recovery ability of composite-modified asphalt;
- The frequency sweep test results were closely related to the softening point (*T_soft_*), viscosity (*η*_135_), mixing temperature (*T_mix_*), and compaction temperature (*T_com_*) of GTRSA. The creep recovery and irrecoverable creep compliance of GTRSA were closely related to the indexes of the GTR content, penetration (P), cone penetration (CP), CPI, and ductility (D), as well as 2S2P1D model parameters (k). In contrast, the relationship between the frequency sweep test outcomes and the MSCR test outcomes showed a weak linear correlation;
- GTR has an obvious enhancement effect on SBS-modified asphalt, but the excessive mixing and compaction temperatures of GTRSA still need to be improved by adding some warm mixing agents and adopting other methods. In addition, it is advisable to further explore the modified mechanism of GTRSA by combining various analytical and experimental methods, as well as to establish the function model between these indicators. These things considered, it is recommended to further study the road performance of GTRSA mixtures, which can provide an idea for developing environmentally friendly long-life asphalt pavements and building a resource-recycling society.

## Figures and Tables

**Table 1:** Technical properties of SBS-modified asphalt.

Test Items	Unit	Values	Standard Values	Specification
Penetration at 25 °C	0.1 mm	68	60~80	T0604-2011
Softening point T_R&B_	°C	71	≥55	T0606-2011
Ductility at 5 °C and 5 cm/min	cm	38	≥30	T0605-2011
Solubility	%	99.4	≥99	T0607-2011
Storage stability	°C	1.5	≤2.5	T0661-2011
Elasticity recovery at 25 °C	%	87	≥65	T0662-2000
After rolling thin-film oven test (RTFOT), according to T0610-2011				
Mass loss	%	0.16	±1.0	T0610-2011
Residual penetration ratio at 25 °C	%	72	≥60	T0610-2011
Ductility at 5 °C	cm	27	≥20	T0610-2011 and T0605-2011

**Table 2:** Properties of ground tire rubber.

Test Items	Unit	Values	Standard Values
Density	g/cm^3^	1.18	1.1–1.3
Metal content	%	0.038	<0.05
Moisture content	%	0.32	<1
Fiber content	%	0.43	<1
Ash content	%	4.5	≤8

**Table 3:** The fitting results of the 2S2P1D model and the Arrhenius equation.

GTR Content	2S2P1D Parameters’ Fitting Results							Arrhenius Equation Δ*E_a_* (kJ/mol)
	k	h	μ	β	τ	G_0_ (kPa)	G_m_ (kPa)	
0%	0.724	0.731	3.560	259.2	0.0006	0	1050	114.173
5%	0.657	0.704	4.182	2472.3	0.0006	0	1050	115.981
10%	0.631	0.666	3.589	2054.9	0.0006	0	1050	116.547
15%	0.574	0.624	3.518	2042.8	0.0006	0	1050	116.612
20%	0.538	0.585	3.591	2010.4	0.0006	0	1050	117.561

## Data Availability

Some or all data, models, or codes that support the findings of this study are available from the corresponding author upon reasonable request. The data used to support the findings of this study are available from the corresponding author upon request.
